# The Neural Mechanism Underlying Cognitive and Emotional Processes in Creativity

**DOI:** 10.3389/fpsyg.2018.01924

**Published:** 2018-10-31

**Authors:** Simeng Gu, Mengdan Gao, Yaoyao Yan, Fushun Wang, Yi-yuan Tang, Jason H. Huang

**Affiliations:** ^1^Department of Psychology, School of Medicine, Jiangsu University, Zhenjiang, China; ^2^Institute of Emotion, School of Psychology, Nanjing University of Chinese Medicine, Nanjing, China; ^3^Department of Neurosurgery, Baylor Scott & White Health, Temple, TX, United States; ^4^College of Medicine, Texas A&M HSC, Temple, TX, United States; ^5^Department of Psychological Sciences, Texas Tech University, Lubbock, TX, United States; ^6^Center for Advanced Study in the Behavioral Sciences, Stanford University, Stanford, CA, United States

**Keywords:** creativity, cognition, basic emotions, monoamine, core affect

## Abstract

Creativity is related to both cognition and emotion, which are the two major mental processes, interacting with each other to form psychological processes. Emotion is the major driving force of almost all creativities, sometimes in an unconscious way. Even though there are many studies concerning the relationship between creativity and cognition, there are few studies about the neural mechanisms of the emotional effects on creativity. Here, we introduce a novel model to explain the relationship between emotions and creativities: *Three Primary Color model*, which proposes that there are four major basic emotions; these basic emotions are subsided by three monoamines, just like the three primary colors: dopamine-joy, norepinephrine-stress (fear and anger), and serotonin-punishment. Interestingly, these three neuromodulators play similar roles in creativity, whose core features are value and novelty (surprise), like the characteristics of the core features of basic emotions (hedonic value and arousal value). Dysfunctions of these neuromodulators may be the reasons for both psychopathology and creativity, in that they can change the thinking styles such as novelty seeking behavior, hyper-connectivity of brain areas, and/or cognitive disinhibition to induce both creativity and psychopathology. This new model will not only help researchers understand the dynamics of basic emotion elements, it can also bring an entirely new perspective into the relationship between psychopathology and creativity.

## Introduction

Creativity is regarded as a multidimensional entity, which is related to both cognition and emotion: the two major mental processes, which interact with each other to form psychological phenomena (Sahin et al., 2016; He et al., 2018). The relationship between creativity and cognition (or intelligence) has been of great interest to researchers since the late 1900s, and the major theoretical and practical findings are that creativity and intelligence are overlapping constructs; intelligence is a necessity but not a sufficient condition for creativity. In addition, some psychologists found that the correlation between creativity and intelligence is very low; some researchers believe that extremely high intelligence negatively affects creativity. Therefore, emotion may play an even more important role in the process. The pervasive idea that creativity is intricately linked to emotion is due to the fact that individuals with affective disorders often exhibit extraordinary levels of creativity in various spheres of life (Holm-Hadulla, 2013; Leung et al., 2014; MacCabe et al., 2018), especially in some famous artists, for example Goethe, the poet (Holm-Hadulla et al., 2010; Holm-Hadulla, 2013). The reason may be that emotion is the major driving force for almost all creativities (Damasio and Carvalho, 2013). According to insights from Freud and Weber, it was found that people could produce more creative artwork such as sculptures or poems when they were forced to suppress their anger, possibly due to sublimation (Kim et al., 2013). Emotion can also affect creativity through obsessive thinking, which is often associated with childhood adversity (Arnsten, 2007; Thomson and Jaque, 2018). Although people with early exposure to childhood adversity experience greater negative effects, they are also endorsed with positive creative performance experiences (Thomson and Jaque, 2018). Creativity, in turn, may be used to modulate negative emotions. Results showed that flexibility, creativity, risk-taking, and complexity are negatively correlated with anxiety and that insight reappraisal can induce insight experience, enhance cognitive changes, and reduce negative emotional responses (Chiu et al., 2018). It is not surprising that creativity plays an important role in reappraisal, which is a well-recognized and widely adopted emotional regulation strategy (Wu et al., 2017). However, even though creativity is thought to be closely related to emotion, there are few studies about its dependence on emotion because of the complexity of emotional studies.

Emotion may be involved in the process of creativity through other “names,” such as unconscious thought (Friis-Olivarius et al., 2017), motivation (Oriol et al., 2016), or personality (Feist, 1998; Chiu et al., 2018). For example, Wallas (1926) studied the cognitive process of creativity (Wiggins et al., 2015) and identified four parts in sequence: (1) preparation, in which the creative goal is identified and considered; (2) incubation, during which conscious attempts at creativity are not made; (3) illumination, the moment of enlightenment when an idea appears in conscious awareness, sometimes called the “Aha!” moment; and (4) verification, in which the new idea is applied (Wiggins et al., 2015). Ward (2003) described incubation as a time when unconscious behavior takes over, which allows for unique connections to be made without consciously trying to make logical order out of the problem. Furthermore, Helie and Sun (2010) proposed a unified framework of explicit-implicit interaction theory for understanding creativity. This theory analyzed five basic principles between explicit thinking and implicit thinking and concluded that creativity encompasses both conscious and unconscious incubation and insight. Emotion can affect creativity through personality too. For example, some researchers took the social-personality approach to measure creativity, like self-confidence, aesthetic orientation, risk-taking, or independent thinking. The most important parts of personality associated to creativity are related to emotion, such as openness, self-acceptance, hostility, and impulsivity.

In reality, emotion is never absent in all the four processes, but how emotion is involved is still a complicated issue. One of the reasons is explained by what Damasio said, emotion is one of the least-studied biological phenomena (Damasio and Carvalho, 2013). Wallas considered creativity to be a legacy of the evolutionary process, which allowed humans to rapidly adapt to rapidly changing environment. Simonton (1999) provided an updated perspective on this view in his book, *Origins of genius: Darwinian perspectives on creativity*. Similarly, basic emotions are genetically hardwired and highly conserved throughout evolution, and these emotions exhibit certain functional and adaptive properties that are shared across a wide phylogenetic range. Here, we explore the relationship between emotions and creativity by studying basic emotions. According to our previous studies, there are four basic emotions: joy, sadness, fear, and anger (Gu et al., 2016). These four basic emotions are the primary emotions, which we coined as the **Three Primary Color model**. In this model, we put the three monoamine neuromodulators in a plane: dopamine-joy, norepinephrine-surprise, and serotonin-dislike. This model is very simple, and it can be used as a tool to investigate the dynamics of basic emotions, the etiology of affective disorders such as depression, and their relations with creativity.

## The Basic Emotions

The main reason for the lack of studies about emotion in creativities may be that emotion is a rather complicated subject. To make complicated things simple, the easiest way to study emotion is possibly by studying the basic emotions. The central idea of basic emotion theory is that human nature constitutes a group of qualitatively distinct emotions (Russell, 2006). Basic emotions are thought be innate and universal and have evolved through their adaptive value with fundamental life tasks (Ekman, 1992); similarly, most creative art forms can find their evolutionary origins (Wiggins et al., 2015). Ekman (1992) proposed that basic emotions have a number of characteristics, which distinguish one emotion from another, such as universal signals, distinctive physiology, and automatic appraisal influenced by both ontogenetic and phylogenetic past. A summary of the studies about basic emotions were shown in a paper by Clore and Ortony (2013), which shows that most studies recognize six **classic** basic elements of emotion: happy, surprise, afraid, disgust, angry, and sad. Interestingly, a recent paper studied people’s facial expressions, and the research found that disgust and anger shared a wrinkled nose and fear and surprise share raised eyebrows (Jack et al., 2014). Therefore, it is concluded that we humans have four **basic** elements of emotion: happy, sad, fear/surprise, and angry/disgust, and these four emotions are the basic building blocks from which we develop our modern, complex, and emotional stews (Jack et al., 2014). Consistently, several other papers also proposed four basic elements for emotion: fear, anger, happiness, and sadness (Gu et al., 2015, 2016; Wang and Pereira, 2016; Zheng et al., 2016).

## Core Affects

**FIGURE 1:**
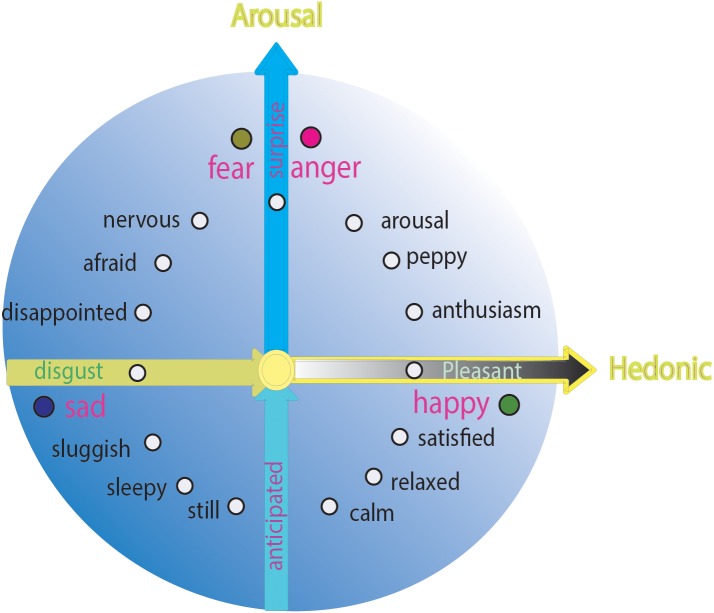
Circumplex model of emotions. One example of self-report datum about circumplex model. All emotions, including the basic emotions can find their locations in the circle of the circumplex.

The best way of studying basic emotions is to place them in dimensions. Russell and Barrett referred to several theoretical emotional studies and concluded that all basic emotions can be arranged in a circumplex (Weierich et al., 2010). The circumplex is defined as a circular arrangement of basic emotions around two independent, bipolar dimensions: hedonic (pleasure–displeasure) value and arousal (rest-activated) value. These two dimensional features are taken to be essential features of all emotions (Gu et al., 2016) and can be named as “core affects” (Russell, 2003; Salzman and Fusi, 2010). The horizontal dimension of the circumplex is the core affect *hedonic value* and the vertical dimension is *arousal value* (Figure 1; Posner et al., 2005). The different location of each emotion means that different emotions have different “amounts” of *hedonic* (including *pleasure–displeasure*) *value* and *arousal* (*rest-activated*) *value*, which are characteristic parameters to define basic emotions.

Many different names have been given to the horizontal dimension, such as liking, valence, hedonic tone, and many other identical items, since Wundt first introduced the dimension (Gu et al., 2015). The arousal dimension has been given to the vertical dimension, for it is related to the arousal states of the body. It depends on whether something happens in unexpected ways or not (Zheng et al., 2016). Similar to what Barrett (2006) proposed, “Arousal is associated with the uncertainty regarding whether a stimulus will predict threat of reward, the need to pay more attention to a stimulus of importance, or urgency to engage in active coping”.

**FIGURE 2:**
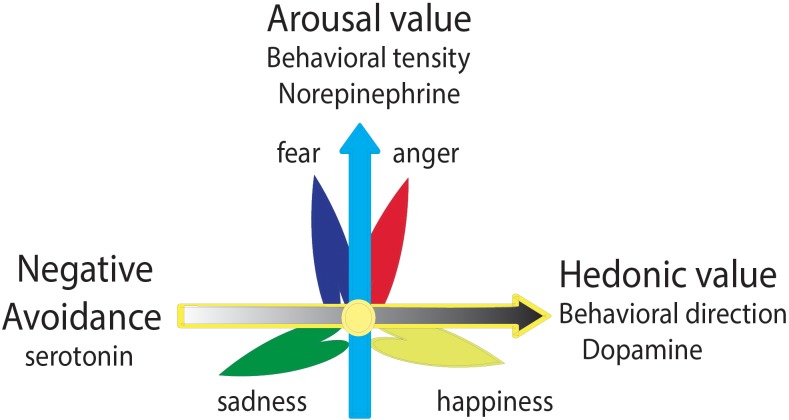
Two dimensional model of basic emotions. Emotions can be induced by two affective qualities of a stimulus: the valence of the stimulus and the way the stimulus occurs. Horizontal dimension shows the valence of the stimulus, which means the hedonic quality of a stimulus or situations when they fit into personal needs (pleasant–unpleasant). Vertical dimension shows how the way a stimulus or situations occur, which means unexpected or uncertainty.

These core affects (valence and arousal) of emotions are due to features of stimuli (the *hedonic value and safety value*) (Zheng et al., 2016; Figure 1). According to appraisal theory of emotion, emotions result from people’s interpretations and explanations of their circumstances. Arnold wanted to “introduce the idea of emotion differentiation by postulating that basic emotions such as fear, anger, and joy could be distinguished by different excitatory phenomena.” In her pioneering studies about “cognitive theory” in the 1960s, Arnold specified that the first step in emotion is an appraisal of the situation. The individual has evolved an evolutionary safety check for the stimulus when it first arrives. If the individual senses danger, he will be scared and will fight or flee for survival. Later, if the individual feels it is safe, he will have a secondary check to see if it fits the individual’s need, then the organism will be happy or sad. In the circumplex, the locations of the four basic emotions (happy, sad, fear, and angry) are very typical: fear and anger are on top of the vertical dimension, whereas happiness and sadness are on the two opposite sides of the horizontal dimension (Figure 1). These typical locations of the four basic emotions suggest that the four basic emotions have different parameters: happiness and sadness are due to the hedonic value of a stimulus (needs), whereas fear and anger depend on the safety value of a stimulus (safety needs) (Gu et al., 2016). These typical features may be the reasons for them to be basic emotions. Complex emotions are mixed with basic emotions, with different amounts of hedonic value and safety value of a stimulus; for example, surprise (fear or anger) + happiness can induce enthusiasm (Arnott and Elwood, 2009) or surprise (fear or anger) + sadness may induce disappointment (Figure 2).

## Neuromodulators for Basic Emotions

With the development of drugs for affective disorders, catecholamine and serotonin have been regarded as the neural substrates for emotion, ever since the 50–60s of the last century (Schildkraut and Kety, 1967). Later on, dysfunctions of the monoamine system proved to be the substrates of many mental disorders such as depression, post-traumatic disorders, and attention-deficit hyperactivity disorder (Dougherty et al., 1999). Most of the first-line antidepressant and anti-psychotic drugs target the monoamine system. Monoamine neuromodulators have a big advantage to work as substrates for the basic emotions: they affect both the peripheral nervous system and the central nervous system. The brain areas that release the monoamine are the ventral tegmental area, locus coeruleus (LC), and raphe nuclei. These monoamine producing systems project their axons and release these neuromodulators diffusely and widely throughout the cerebral cortex (Lovheim, 2012).

**FIGURE 3:**
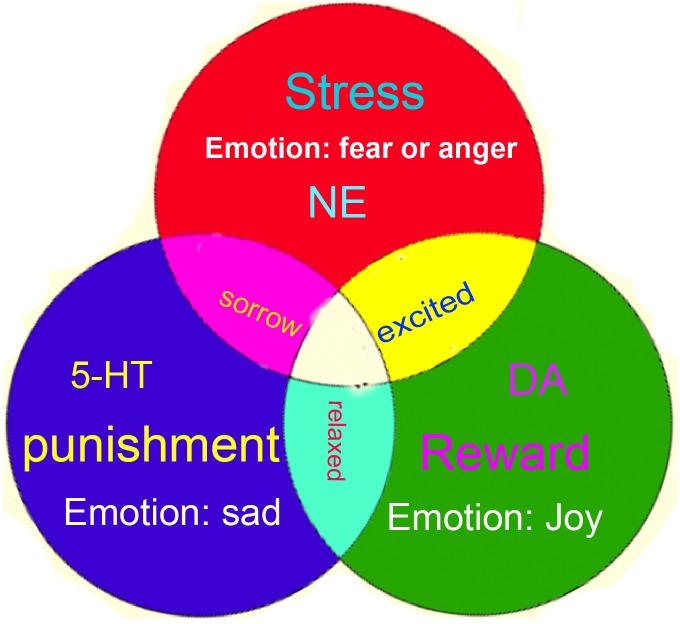
Three primary color models of basic emotions. The four basic emotions: fear-anger, joy, sad, or three core affects are subsided by the release of three monoamine neuromodulators (norepinephrine-stress, dopamine-reward, and serotonin-punishment).

Monoamine neuromodulators are proposed to be substrates for the basic emotions: dopamine-pleasant, serotonin-displeasant, and norepinephrine-arousal (Lovheim, 2012; Gu et al., 2016; Wang and Pereira, 2016), like the three primary colors (Figure 3). However, even though numerous studies from different fields support the notion that all three monoamines are involved in affective diseases, their effects are still mixed. For example, antidepressant drugs affect almost all the monoamine neuromodulators and are also used for almost all affective diseases such as anxiety, phobia, depression, etc. There is a need to improve the conceptualization and classification of the emotional states and the neuromodulators. Here, we try to differentiate their functions in controlling emotion and behavior, and a new hypothesis about monoamines and emotions is introduced: three monoamine neuromodulators underlie the three core affects (dopamine-reward, serotonin-punishment, and norepinephrine-surprise); they work together to make different basic emotions, like the three primary colors. The dopamine (DA) system has been proved to be involved in reward (Bressan and Crippa, 2005; Haber and Knutson, 2010; Diana, 2011), the noradrenaline system has been related to the “fight or flight” responses at stressful events (Herrmann et al., 2004; Benarroch, 2009), and the serotonin system seems to be related to punishment (Tops et al., 2009). Their roles in creativities are also important.

### Dopamine-Reward

Brain mesolimbic DA has long been linked to the rewarding processes in the brain ever since Wise (1980) first proposed the hedonic hypothesis of DA and proposed that DA is a signal of stimulus salience, providing the feeling of enjoyment (Bergamini et al., 2016). From then on, numerous studies have supported DA’s role in the rewarding signals for food, sex, and other needs, which are often stimulated by reward seeking behaviors (Frank et al., 2016). Many pharmacological and behavioral studies on intracranial self-stimulation have established the important role of the medial prefrontal DA system in reward behavior. As a result, the term DA has been widely used synonymously with reward or happiness.

### Norepinephrine – Surprise

Novelty, either real or perceived, to the organism, can induce robust norepinephrine release, and the brain norepinephrine system is well known to be activated by surprise or novelty (Morilak et al., 2005; Gu et al., 2015, 2016). Following exposure to novel signals, norepinephrine is released from the LC to the brain cortex and to almost all other limbic areas, such as the hypothalamus (Birnbaum et al., 1999; Leonard, 2001; Morilak et al., 2005; Barbieri et al., 2015). Even though the LC is very small, the axons of these neurons project to essentially the whole brain and potentially influence the entire nervous system. Both electrophysiological and neurochemical studies have shown that the brain LC is robustly activated by unexpected events (Ma and Morilak, 2005; Bott-Flügel et al., 2011), and norepinephrine release is determined by the salience of the stimulus. Novelty is a key feature of creativity (Wiggins et al., 2015), and it is proposed that the attribution of creativity entails the attribution of novelty; also, human creative drive is the search for novelty (Wiggins et al., 2015).

### Serotonin – Punishment

Serotonin plays a critical role in a wealth of psychiatric conditions, such as depression, manic anxiety, and obsessive compulsions. However, despite the importance of serotonergic pharmacotherapies, particularly selective serotonin reuptake inhibitors, their roles in normal emotion are still mysterious (Dayan and Huys, 2008). More than 20 years ago, Deakin and Graeff hypothesized that different serotonin pathways act in response to aversive stimuli, such as through opposition of DA (Dayan and Huys, 2008), and the dysfunction of these pathways contributes to the pathophysiology of anxiety and affective disorders (Deakin and Graeff, 1991). Later, many studies have related serotonin to punishment, for example, Robinson showed that “serotonin is critical for punishment-induced inhibition” (Crockett et al., 2009, 2012; Robinson et al., 2012). Dayan concluded that “At a global level, serotonin is richly involved in the behavioral neuroscience of punishment and threat” (Dayan and Huys, 2009). As for creativity, the aesthetic is sometimes comfortable to the author, but sometimes it is utterly incomprehensible and even offensive to some observer, which might be a punishment aspect of the art.

## Relationship With Creativity

Pleasure and arousal are the core affects of emotion. Izard (2009) suggested, “core affect” is not, in and of itself, a mental state of emotion; instead, it is just a feature of emotion. By his report, arousal is associated with the uncertainty regarding whether a stimulus will predict threat of reward, the need to pay more attention to a stimulus of importance, or an urgency to engage in active coping. Emotions are both tendencies of actions as well as consequences of actions; therefore, actions, including creativity, have many elements, which are similar to the features of emotion. For example, the core features of emotions (arousal and reward and/or punishment) are similar to the core features of creativity (novelty and value).

### Value and Novelty Are Two Basic Quantities of Creativity

**FIGURE 4:**
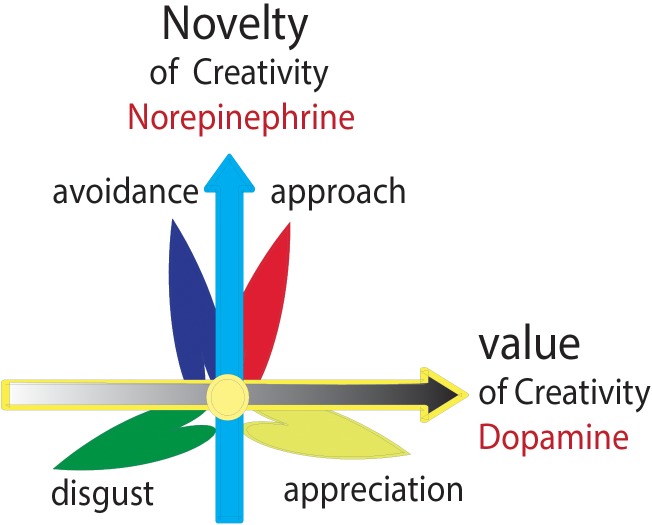
Two features of creativity. Value and novelty are two basic quantities of arts, similar to hedonic value and arousal value of core affects, which are subsided by the release of two catecholamine neuromodulators (norepinephrine-stress, dopamine-reward).

By combining these emotional studies, we set out to explore the relationship between basic emotions and creativity. Konrad Lorenz and Nikolaas Tinbergen, the founders of modern neuroethology, revealed that **animal behaviors are expressions of some innate drives or instincts** (Asahina, 2017). It is proposed that animal behaviors, from egg-rolling in geese to the honeybee waggle dance, are executed by genetically programmed neural circuits that are triggered by specific sensory stimuli (Perry et al., 2016). Creativity is a type of human behavior, which is an expression of human innate states. In Western societies, “creativity” is most commonly used to refer to the embodied cognitive process that gives rise to pieces of music, sculptures, paintings, poems, and other accomplishments that are taken or presented as art. Value and novelty are the two basic simple quantities of art (Wiggins et al., 2015; Figure 4), and they are two major characteristics of creativity. Also, it is certainly true that creative people tend to seek novel stimuli over familiar or simple stimuli (Reuter et al., 2005). This is similar to the core affects of emotions (hedonic value and arousal value).

### Value

The core affects of pleasure and arousal are due to stimulation effects on the subjects. Consistently, value and novelty of creativity are not two discrete kinds of creativity; instead, they are relations between observers and the created artifact (Wiggins et al., 2015). Value is dependent not only on the observer but also on the context in which the observation is made (Wiggins et al., 2015). Value is not only a characteristic of creativity, it is also present in many more pursuits apart from the artistic ones mentioned above (Wiggins et al., 2015). A prime example is mathematics, where the creation of the proof of a theorem is more highly valued if it is “elegant,” according to the principles of the particular branch of mathematics to which it applies. Evolutionarily, **value** is anything that is elegantly coping with the situation. This is consistent with Lazarus’s appraisal theory of emotions (Wang et al., 2018).

**FIGURE 5:**
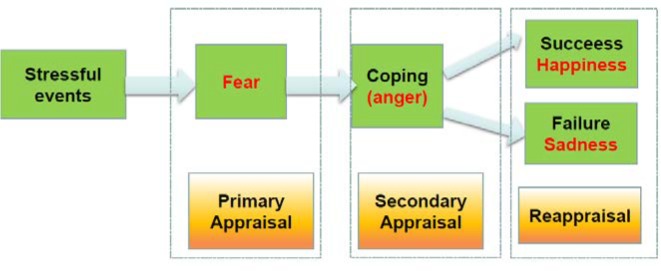
Stress induced emotion flow. Like Lazarus suggested, there are two kinds of appraisals, the primary appraisal is to find the fearful situation, and the secondary appraisal to find the ability to cope with the situation. Fear is due to the first appraisal, and anger is due to the secondary appraisal. And afterwards, there will be reappraisal, which includes emotion based rethinking positively/negatively about the situation. Stress–Fear–Response (anger)– Consequences (happiness or sadness) constitute the emotional flow in our everyday lives. Similarly, creativity value is due to successfully coping with the situation.

Richard Lazarus followed closely with Magda Arnold in the research of emotions through cognitive appraisal. Lazarus specified two major types of appraisals: (1) primary appraisal, directed at the establishment of the significance or meaning of the event to the organism and (2) secondary appraisal, directed at the assessment of the ability of the organism to cope with the consequences of the event. These two types of appraisals go hand in hand as one establishes the importance of the event. The first appraisal is related to harm and threat and induces fearful emotion to motivate avoidance and withdrawal. The second appraisal is conscious and concerned with coping (Lazarus, 1999; Zheng et al., 2016). Both fear and anger are due to unexpected stressful events (also see Figure 5; Zheng et al., 2016); fear is associated with feelings of uncertainty, whereas anger is associated with coping with stressful situations (Moons et al., 2010). After coping with stressful situations, Lazarus proposed a certain type of cognitive reappraisal processes, which included positive reappraisal (happy or rewarding emotions will be induced) and negative reappraisal (sad or punishing emotions will be induced and negative reappraisal (sad or punishing emotions will be induced) (Aldwin, 1994; Lazarus, 1999). If the organism can cope successfully with the stressful situation, the organism will then show positive emotions and be happy. Otherwise, the organism will experience negative emotions and be sad (Wang et al., 2017). Therefore, we propose that the emotion **joy is due to positive reappraisal: coping successfully**. This can also be reflexed on the value of creativity, in that creativity is the smartest way to deal with difficult situations in life. Altogether, *the happy emotion and creativity value are both due to the successful coping with the situation*.

### Novelty

Novelty is another key feature of creativity. In Western culture, as we have articulated above, the attribution of creativity entails the attribution of novelty–various authors have argued that the human creative drive is the search for novelty (Justin and Sloboda, 2010). Huron proposed that it is appropriate for an animal to be alert and prepared for fight or flight in the face of novel circumstances, because the outcome of a novel experience is sometimes dangerous (Huron, 2006). Research based on physiological and behavioral measures in human responses to live music also found that the unexpectedness value of pitch can induce a significant part of the variance in physiological measures (heart rate, skin conductivity, etc.) that correspond with arousal (Salzman and Fusi, 2010). This constitutes evidence that unexpectedness in music correlates with arousal in listeners (Wiggins et al., 2015). Huron suggested that tension, thus, stimulated by expectation is in large part responsible for affects stimulated by Western music, whose emotive content is frequently in theory conceived as an ebb and flow of tension of various types. This affective experience is highly valued and, altogether, more subtle and dynamic than the common labeling of emotional analysis of music as “tender,” “sad,” etc. (Justin and Sloboda, 2010). Weiss et al. found that when nightingales heard a playback consisting of song types with branch transition patterns, they responded with song types that had bottleneck transition patterns. Conversely, when they heard song types with bottleneck transition patterns, they responded with song types that tended to be branching transitions in their population; that is, they responded with the unexpected (Justin and Sloboda, 2010).

## Neuromodulators for Creativity

Monoamine neuromodulators are substrates of basic emotions. So, what are their potential roles in creativity?

### Value-Dopamine

The hedonic hypothesis of DA postulates that DA in the brain plays a critical role in the subjective pleasure associated with positive reward. However, several complications in recent DA studies have opened this theory to debate and reexamination (Berridge and Kringelbach, 2015). The incentive salience hypothesis was recently introduced, which suggested that the major function of DA is not only to mediate the unconditioned pleasures from food, sex, and drugs but is also linked to the anticipatory, preparatory approach or the coping phases of reward behavior (Sandoval and Seeley, 2017) and in the relations of DA neuron to the reward outcome (Schultz et al., 1997). This hypothesis is highly consistent with Lazarus’s (1999) reappraisal theory about happiness or sadness: *the happy and sad emotions are related to the success or failure to cope with stressful situations* (Wang et al., 2018).

This is really the case for DA neurons. Schultz screened the controlling process in great detail and found that DA release is the highest during the learning process. He suggested the name *predication error* for the learning process (Schultz et al., 1997; Eshel et al., 2016). It appears that learning is driven by deviations or “errors” between the predicted time and amount of rewards. Schultz et al. (1997) proposed that DA encodes expectations about external stimuli or reward, especially when it is uncertain or there is a deviation or error (predication error). *Therefore, DA release is not determined by rewarding stimuli or aversive stimuli. Instead, it is determined by the outcome of whether the coping process is successful or not*. Altogether, the DA system is a reward process, which is determined by the coping process (Cabib and Puglisi-Allegra, 1994; Spreux-Varoquaux et al., 2001).

The value of an art is the interaction between production and (probably, at least initially, introspective) evaluation by an artist, and then by a social community, that identifies relative value and relative novelty, of both the artifact and the way it was made. Margaret Boden makes another, perhaps more tractable, distinction between psychological creativity–the act of generating an artifact that is novel and of value to an individual–and historical creativity–the act of generating an artifact that is novel and valued in historical terms. Consistently, reward will in turn affect creativity. It is found that creativity-contingent rewards tend to increase creative performance, and these rewards are more positively related to creative performance when individuals are given more positive, contingent, and task-focused performance feedbacks and are provided more choice. In contrast, performance-contingent or completion-contingent rewards tend to have a slight negative effect on creative performance (Byron and Khazanchi, 2012).

### Novelty-Norepinephrine

Stress results from real or perceived threat to the homeostasis or well-being of the human being (Zheng et al., 2016), and it is due to the uncertainty about the situation. Stress can activate the norepinephrine/locus coeruleus (NE/LC) system, which induces fight or flight behaviors and fear and anger emotions. Darwin descried two primary mechanisms of selection as the driving forces of biological evolution, natural selection, and sex selection. The critical elements for natural selection are variations in traits, while sexual selection can be viewed as a special case of natural selection, which acts on an individual’s ability to mate, such as fighting. Biologists have investigated a variety of modes of sexual selection for mate choice. The simplest selection will be for a character that provides a direct benefit, such as if a female bird chooses a male whose genes produced a tail of the optimal size for fighting (Justin and Sloboda, 2010). Creative behavior can also result in sexual selection, for example, learning to weave a beautiful nest by the male weave-birds can give them a better chance to mate. Altogether, emotion can drive creativity through an attentional style driven by novelty salience (Carson, 2011). Internal rewards for seeking novelty may provide creative people with intrinsic motivation and intellectual curiosity (Schweizer, 2006). In addition, creative people tend to seek novelty; therefore, the character of novelty-seeking may be an incentive for creative work (Carson, 2011).

### Serotonin-Inhibition

When an individual fails to cope with a stressful situation, serotonin will be released (Chaouloff et al., 1999). Serotonin is correlated positively with aversion and negatively with reward. This effect can also be demonstrated by serotonin’s analgesic properties, and in fact, selective serotonin reuptake inhibitors taken chronically have an important role in the clinical management of chronic and neuropathic pain (Dayan and Huys, 2008). Numerous studies have found that serotonin is linked to aversive conditioning, reward suppression, and behavioral inhibition (Dayan and Huys, 2009). Therefore, serotonin has a major behavioral effect on suppression, inhibition, or freezing. Decreased inhibition is associated with increased creative achievement (Carson et al., 2003). Decreased inhibition increasing creativity may work through the disinhibition of hyperconnectivity, which is an abnormal neural linking of brain areas that are not in general functionally connected (Carson, 2011). Hyper-connectivity has been reported in the brains of highly creative subjects during creativity tasks, which may provide the neurological mechanism for remote associations between stimuli that are the basis for creative thought.

## Creativity and Psychopathology

Despite the fact that creativity is a highly valued trait and viewed as an aspect of self-actualization, the possibility of creative people to suffer from psychosis has been noted ever since the ancient times. Many biographies and empirical studies have found that creativity has been associated with psychopathology. For example, it was found that more than 80% of the writers had suffered from mood disorder, which is four times higher than that of the control (Carson, 2011). The major reason is that *shifts of mental states associated with mood dysfunction can facilitate creativity*. Genetic vulnerability factors related to the functioning of DA and serotonin in the prefrontal brain and subcortical brain are the major reasons that predispose certain people to experience altered mental states (Carson, 2011). These altered emotional states may manifest in these people as severe psychopathology or as creative ability (Carson, 2011). Molecular genetic studies have begun to hone in on a set of genes, most of them are related to NE, DA, and serotonin transmission that are associated with both creativity and mood disorders (Carson, 2011). For example, several genes that are related to DA, including *DRD4*, *SLC6A3*, and *Taq1A*, have been linked to both risks of schizophrenia and bipolar disorder as well as novelty seeking in creativity. Catechol-O-methyltransferase (COMT), an enzyme responsible for degrading catecholamine, including DA and NE, has been implicated in schizophrenia and many mental disorders, and it has also been associated with creativity.

## Conclusion

Creativity is considered a positive personal trait; however, highly creative people often demonstrate elevated risks for certain forms of psychopathology, such as mood disorders. How emotion affects creativity is still not entirely clear. Here, we offered a model to explain the relationship between creativity and emotion. This model, supported by recent findings from neuroscience and molecular genetics, suggested that the hyper-functions of neuromodulators (or hypo-function) confer the emotional pathology and also enhance creative ideation. These dysfunctions of neuromodulators might induce both mood disorders and creativity, via cognitive disinhibition, attentional style driven by novelty salience, and neural hyper-connectivity that might increase associations among disparate stimuli.

The mechanism of this model lies in that neuromodulators, including NE and DA, are the neural basis for both creativity and basic emotions, and their dysfunction can offer motivation and novelty seeking as well as hyper-connectivity for the brain. Structurally, values and novelty, determined by NE and DA, are key features for both creativity and basic emotions. Functionally, emotion flow follows a pathway: fear–anger–joy–sadness at stressful situations, which is similar to the process of creativity (which follows a procedure of preparation, incubation, illumination, sometimes called the “Aha!” moment, and verification). Altogether, this model will not only be helpful in better understanding the dynamics of basic emotions, it can also bring a brand new perspective in creativity.

## Author Contributions

SG, FW, and JH designed the work. SG, FW, JH, and Y-yT did the writing. MG and YY helped with drafting the figure and did some revisions for the work.

## Conflict of Interest Statement

The authors declare that the research was conducted in the absence of any commercial or financial relationships that could be construed as a potential conflict of interest.
